# Acetozone as an Intestinal Antiseptic in Typhoid Fever

**Published:** 1903-07

**Authors:** 


### Acetozone as an Intestinal Antiseptic in Typhoid Fever.

Frederick G. Harris, Chicago, (*Therapeutic Gazette,* March, 1903,) reports 128 cases of typhoid treated in Cook County

Hospital, Chicago, with acetozone. The cases first admitted seemed to indicate that the epidemic was of a mild form, but later the disease proved to be of a severe type and complications were numerous. The author obtained the most satisfactory results with aqueous solutions of 15 grains to the quart, which the patients were urged to use very freely to quench the thirst, while in addition four or six fluid ounces of the solution were given every four hours as a therapeutic measure. The movements of the bowels were regulated with sodium phosphate or magnesium sulphate. The temperature of the patients, on admission, was high, as a rule. In 117 cases under acetozone treatment the average duration of the fever was 18 days.

The number of recoveries was 117, or 91.4 per cent., while 11 patients died, a mortality of 8.59 per cent.; statistics of the cases of typhoid fever in the same hospital (Cook County) not treated with acetozone show a death-rate of 13.1 per cent. The author is of the opinion that under the acetozone treatment, in favorable cases, the duration of the disease was materially shortened, and the most disagreeable symptoms were ameliorated. He declares that the characteristic fetor of the stools and the peculiar odor of the wards was greatly diminished; there was less stupor and delirium and less tympanites, and the usual diarrhea was checked. An average of 138.12 grains of acetozone was used in each case. Finally, he reaches the conclusion that when cases can be seen during the first week of the attack and large amounts of acetozone given, assisted by a gentle laxative, the temperature will return to the normal in from ten to twelve days.

Four cases of typhoid fever, in which acetozone was employed with satisfactory results, were reported by Charles Emil Brack, Baltimore (*Medical Age*, January 25). In each case the treatment consisted in the use of acetozone in solution. The first three patients, adults, received 30 grains of the drug per diem; the fourth, a child of 4 years, received 8 grains each 24 hours. Prompt recovery occurred in each case.

James Billingslea, Baltimore, (*Atlanta Journal-Record of Medicine*, February, 1903,) reported twenty-five cases of typhoid fever treated with acetozone. The diagnoses were confirmed by board of health examinations. The treatment consisted in clearing the bowels thoroughly by means of calomel. Liquid diet was prescribed and cold or sponge baths were used as occasion required. The special treatment consisted in shaking 15 or 20 grains of acetozone powder with one quart of water, allowing the insoluble residue to subside. The patient was given the clear solution to drink freely, the whole amount of one quart being taken during 24 hours. The writer suggests that one part of the acetozone solution may be mixed with three parts of milk, if thought desirable. The action of acetozone will be materially aided by the use of a mild saline laxative.

He found that the feces soon lost their disagreeable odor by this treatment, and cold baths were required to a much less extent than with other treatment. Furthermore, the nurses universally affirmed that they found patients under this treatment easier to care for. No evil effects were noted from the use of acetozone.

A further contribution to this subject appears from the pen of J. J. Driscoll, Chicago, (*Kansas City Medical Index-Lancet*, January, 1903,) who relates his experience in six cases. He found that acetozone reduces the temperature, shortens the duration of the disease materially, while it does not seem to have any ill effects on the heart. The feces are completely deodorised in 36 to 48 hours, and tympanites rapidly disappear.

